# Supplementary material for: Comparative multiomics analysis of cell physiological state after culture in a basket bioreactor
Source: Sci Rep. 2022 Nov 23;12:20161. doi: 10.1038/s41598-022-24687-4 (PMC9686226; doi:10.1038/s41598-022-24687-4)

8916:1780897:-@NW_023666051.1:1753350:1753466:-@NW_023666051.1:1707047:1707068:-@NW_023666051.

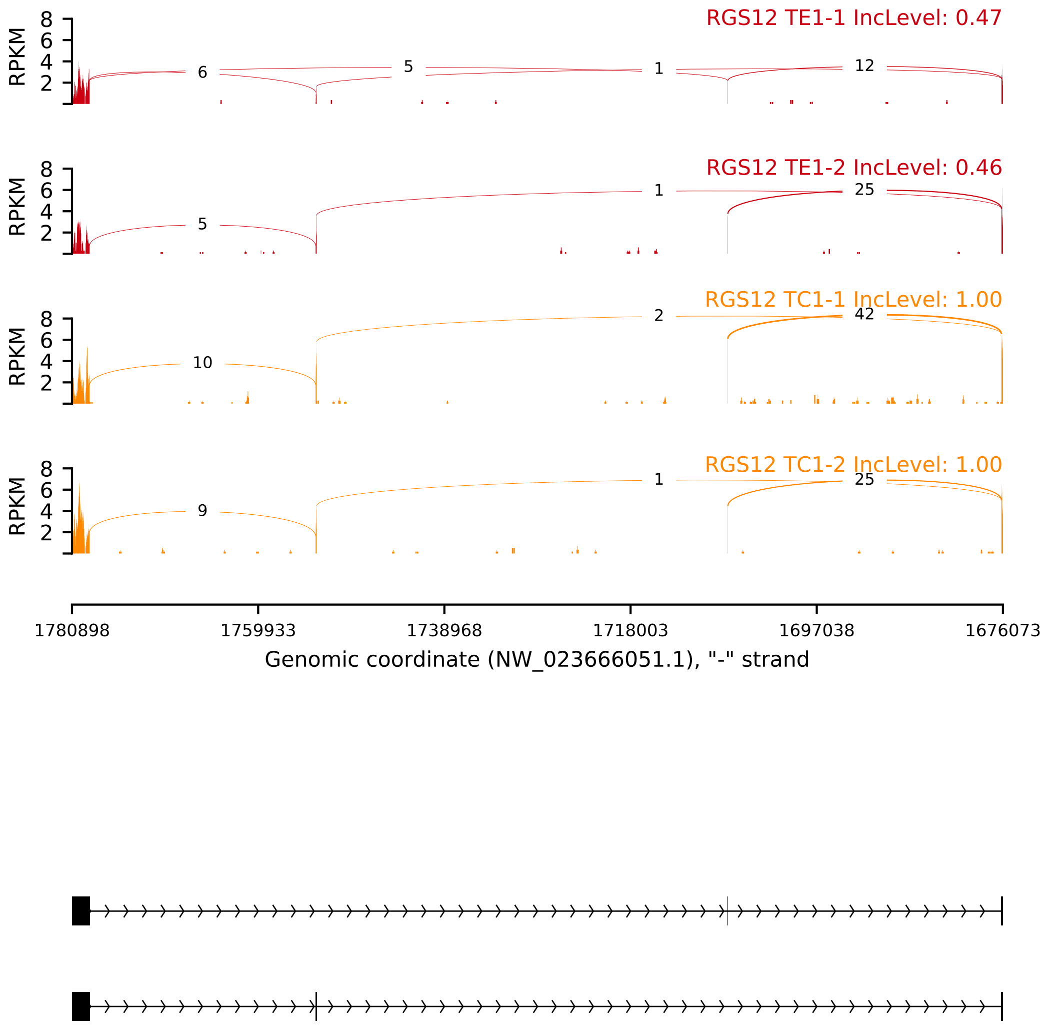

Supplement: Supplementary file 1 — Supplementary Information 1. [file 41598_2022_24687_MOESM1_ESM.zip › raw data/Transcriptomic raw data/7.AS/2.dasplot/TE1vsTC1/MXE/7_RGS12_NW_023666051.1_1778916_1780897_-@NW_023666051.1_1753350_1753466_-@NW_023666051.1_1707047_1707068_-@NW_023666051.1_1676071_1676240_-.pdf]
